# Ataxia-Telangiectasia Mutated is located in cardiac mitochondria and impacts oxidative phosphorylation

**DOI:** 10.1038/s41598-019-41108-1

**Published:** 2019-03-18

**Authors:** Marguerite Blignaut, Ben Loos, Stanley W. Botchway, Anthony W. Parker, Barbara Huisamen

**Affiliations:** 1 Division of Medical Physiology, Department of Biomedical Sciences, Faculty of Medicine and Health Sciences, Stellenbosch University, Tygerberg, 7505 South Africa; 2 Department of Physiological Sciences, Faculty of Sciences, Stellenbosch University, Stellenbosch, 7602 South Africa; 3 Biomedical, Research and Innovation Platform, South African Medical Research Council, Tygerberg, 7505 South Africa; 4 Central Laser Facility, Research Complex at Harwell, STFC Rutherford Appleton Laboratory, Harwell Campus, Didcot, OX11 0QX UK; 5 Oxford Brookes University, Department of Biological and Medical Sciences, Oxford, OX3 0BP UK; 6 Department of Physics, Faculty of Science, Stellenbosch University, Private Bag X1, Matieland, 7602 South Africa

## Abstract

The absence of Ataxia-Telangiectasia mutated protein kinase (ATM) is associated with neurological, metabolic and cardiovascular defects. The protein has been associated with mitochondria and its absence results in mitochondrial dysfunction. Furthermore, it can be activated in the cytosol by mitochondrial oxidative stress and mediates a cellular anti-oxidant response through the pentose phosphate pathway (PPP). However, the precise location and function of ATM within mitochondria and its role in oxidative phosphorylation is still unknown. We show that ATM is found endogenously within cardiac myocyte mitochondria under normoxic conditions and is consistently associated with the inner mitochondrial membrane. Acute *ex vivo* inhibition of ATM protein kinase significantly decreased mitochondrial electron transfer chain complex I-mediated oxidative phosphorylation rate but did not decrease coupling efficiency or oxygen consumption rate during β-oxidation. Chemical inhibition of ATM in rat cardiomyoblast cells (H9c2) significantly decreased the excited-state autofluorescence lifetime of enzyme-bound reduced NADH and its phosphorylated form, NADPH (NAD(P)H; 2.77 ± 0.26 ns compared to 2.57 ± 0.14 ns in KU60019-treated cells). This suggests an interaction between ATM and the electron transfer chain in the mitochondria, and hence may have an important role in oxidative phosphorylation in terminally differentiated cells such as cardiomyocytes.

## Introduction

Ataxia-Telangiectasia (A-T) is a rare, recessive disease characterised by the absence of Ataxia-Telangiectasia Mutated (ATM) protein kinase. The disease results in progressive neurodegeneration, increased incidence of cancer, radio -sensitivity, insulin resistance and cardiovascular disease^1,2^. A recent meta-study revealed that heterozygous A-T carriers, that make up as much as 1.4–2% of the general population^3^, have a significantly increased risk of developing cancer and ischaemic heart disease^4^. Mouse studies have also shown that heterozygous ATM mice suffer from accelerated atherosclerosis development^5^, and that the absence of ATM can lead to structural and functional changes in the heart^6^. Although ATM protein kinase is best known for its role in the signalling and repair of double strand breaks in DNA^7^, available data suggests that the absence of ATM protein kinase can also result in a plethora of metabolic and cardiovascular abnormalities. Its potential importance with regards to the development of insulin resistance and vascular dysfunction has also been highlighted^8^. Studies have shown that anti-oxidative treatment specifically targeting mitochondria can decrease metabolic syndrome in ATM-null mice^5^, and supports the notion that A-T might be a mitochondrial disease^9^. Currently, an emerging role for ATM is being investigated independently from the DNA damage response pathway with regards to its activation in response to mitochondrial oxidative stress^10,11^ and contribution towards oxidative homeostasis in the cytosol^11–13^.

Cardiomyocytes make up approximately 70–85% of all heart cells^14^. Mitochondria comprise approximately 30% of the cardiomyocyte volume^15^ and are essential for the maintenance of healthy cardiomyocytes^16^. Mitochondrial dysfunction leads to increased reactive oxygen species (ROS) production and consequently, oxidative stress. Moreover, dysfunctional mitochondria have been identified as a contributing factor to both insulin resistance and coronary artery disease^17^, as well as various cardiac pathologies including the progression of heart failure^18^.

ATM can act as an important sensor of oxidative stress in cells and regulate redox stress defences^10^, but the underlying mechanisms are still largely unclear. ATM is known to regulate mitochondrial biogenesis and DNA content^19^ and can lead to mitochondrial dysfunction when absent^20^. ATM has also been detected in mitochondrial fractions of normal human fibroblasts, and can be activated in response to mitochondrial uncoupling^21^. *Atm*-deficient cells (ATM^−/−^) show decreased respiratory rates compared to their wild-type counterparts, which can be partially repaired with anti-oxidant treatment^20^. Earlier reports of ATM localisation showed that mitochondrial subcellular fractions of human fibroblast^21^ and hepatocyte cell-cultures are enriched with phosphorylated ATM in response to cytosolic oxidative stress, whilst the fraction containing peroxisomes only contained traces of ATM^22^. Most recently however, ATM was shown to be imported into the peroxisome via the PEX5 peroxisome import receptor in response to oxidative stress^23^ and reacts to peroxisomal ROS to induce autophagy of the peroxisome (pexophagy)^24^. Furthermore, in HeLa cells, ATM localises in the nucleus in response to endogenous mitochondrial oxidative stress, independently of DNA damage response, whilst promoting glucose flux through the pentose phosphate pathway (PPP) and increasing cellular anti-oxidant capacity by increasing glucose-6-phosphate dehydrogenase (G6PD) in bone osteosarcoma cells (U2OS)^11^. Impaired ROS sensing by ATM results in increased glutathione synthesis, possibly due to a decreased flux through the PPP. This reduces NADPH availability for glutathione recycling and places ATM, as a redox sensor, at a critical junction of cytosolic carbohydrate metabolism^11^. The redox pairs, NAD^+^/NADH and phosphorylated NADP^+^/NADPH, play critical roles respectively in cellular redox states during ATP production and ROS production, or lipid, amino acid and nucleotide biosynthesis through glycolysis or the PPP^25^.

Whilst it is well known that ATM resides predominantly in the nucleus of mitotic cells^26^, the protein is found mainly in the cytoplasm of differentiated neuronal cells where it maintains basal metabolic flux^27^. This study sought to establish where ATM is located within terminally differentiated cardiac cells, and if so, whether there are any mitochondrial respiratory or metabolic implications.

We investigated endogenous ATM protein kinase in cardiac mitochondria isolated from male Wistar rats as well as its role in oxidative phosphorylation and effect on NAD(P)H/NADH ratios. Our results show that endogenous ATM is located in cardiac mitochondria from rats under basal conditions, and that direct chemical inhibition of ATM results in decreased oxidative phosphorylation in cardiac mitochondria. We also established that the ATM inhibitor, KU60019, significantly reduced enzyme binding of NAD(P)H/NADH (2.77 ± 0.26 ns compared to 2.57 ± 0.14 ns in KU60019-treated cells), using fluorescence lifetime imaging microscopy (FLIM) in H9c2 rat cardiomyoblast cells.

## Results

### ATM is located on the inner mitochondrial membrane of isolated cardiac mitochondria

**Figure 1:**
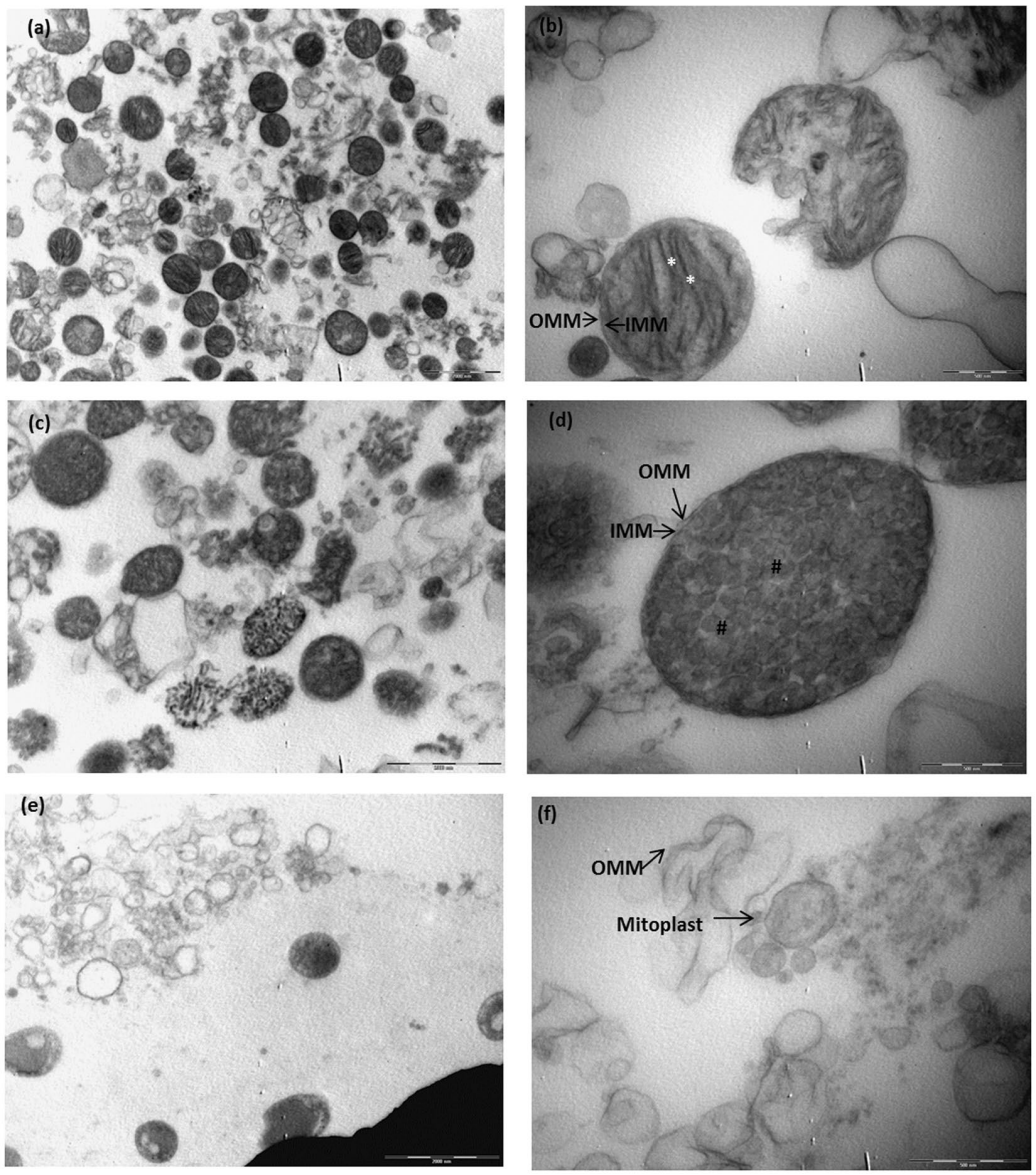
Transmission electron micrograph of mitochondrial and mitoplast preparation. (**a**) Shows an oxidative active mitochondrial preparation and an enlargement of a structurally intact mitochondrion (**b**) where the inner (IMM) and outer mitochondrial membrane (OMM) can be distinguished (arrows) as well as structured cristae (*), seen in panel **b**. (**c**) Shows isotonically swollen mitochondria (M/S buffer) after the addition of 1.2% digitonin quenched at 0 min, and (**d**) an enlargement of mitochondria where a loosening OMM and intact IMM (arrows) as well as a change in cristae structure (round; #) was observed. (**e**,**f**) Represent the mitoplast fraction after 20 min of 1.2% digitonin permeabilisation. Loose OMM fragments were observed as well as round mitoplast structures (arrows). Scale bars represent 2 µm in (**a**,**c**,**e**) and 0.5 µm in (**b**,**d**,**f**).

**Figure 2:**
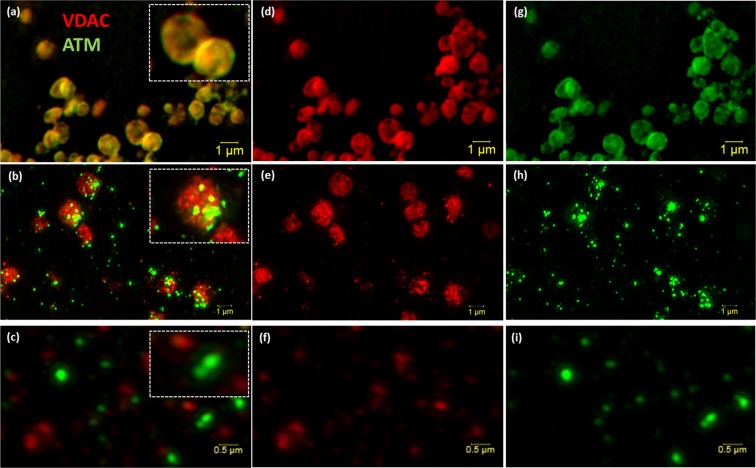
Super resolution structured illumination microscopy (SR-SIM) of isolated mitochondria and mitoplast preparation. M/S-isolated mitochondria (**a**, overlay) was probed with VDAC (**d**; red) and ATM (**g**; green). (**b**) (Overlay of **e**,**h**) shows the permeabilisation of the outer membrane (**e**; red) of M/S isolated mitochondria and formation of mitoplasts directly after the addition of 1.2% digitonin (0 min). (**c**) (Overlay of **f**,**i**) represents the formation of mitoplasts after 20 minutes of 1.2% digitonin treatment. Scale bars represent 1 μm in Panel a,b,d,e,g,h and 0.5 μm in (c,f,i).

Mitochondria were isolated from male Wistar rat hearts with differential centrifugation in either an isotonic isolation buffer (KE buffer; Fig. 1a,b) or in a D-mannitol/sucrose buffer (M/S buffer, Fig. 1c,d). Isolation in an M/S buffer aids the removal of the outer mitochondrial membrane with the mild, ionic detergent, digitonin. The mitochondria were subjected to 1.2% digitonin permeabilisation for 20 minutes (Fig. 1e,f) in order to obtain mitoplasts which consist of the inner mitochondrial membrane (IMM) and matrix^28^. The efficacy of digitonin-based removal of the outer mitochondrial membrane (OMM) was evaluated with transmission electron microscopy (TEM, Fig. 1), prior to being probed with immuno-fluorescent based antibodies against the Voltage-dependent anion-selective channel, VDAC, (OMM marker), TOM20 (OMM marker, Supplementary Material Fig. S1) and ATM with super-resolution structured illumination microscopy (SR-SIM, Carl Zeiss LSM 780) as shown in Fig. 2.

Overlays of the VDAC and ATM labelled images show structurally intact mitochondria (Fig. 2a) and M/S isolated mitochondria directly after the addition of digitonin (Fig. 2b) and 20 minutes (Fig. 2c) of digitonin permeabilisation. This indicates that ATM is not associated with the OMM, nor digested by the digitonin, but rather associated with mitoplasts. This was confirmed with Proteinase K digestion (Supplementary Material Fig. S3). Whereas mitochondrial structure could still be observed prior to digitonin treatment, no mitochondrial structure is seen after digitonin treatment, although clustering or punctate formation of ATM is observed. ATM does not interact with either VDAC (Fig. 2c) or TOM20 after 20 min of digitonin treatment (Supplementary Material Fig. S1).

**Figure 3:**
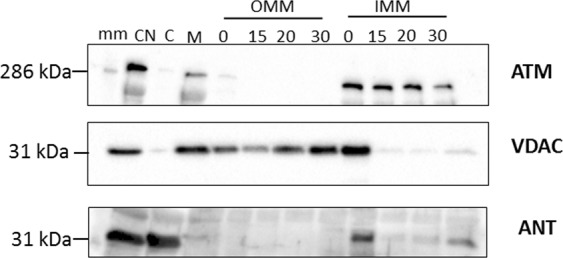
Western blot of subcellular fractionation of the mitochondrial fraction (M) into OMM and mitoplast (IMM and matrix) with 1.2% digitonin. The M/S-isolated mitochondrial fraction was permeabilised with digitonin over time (0 min, 15 min, 20 min and 30 min) and separated into an OMM and mitoplast (IMM and matrix) fraction with differential centrifugation prior to western blotting with antibodies for ATM (350 kDa), VDAC (32 kDa) and ANT (34 kDa). The images were cropped and the uncropped blots as well as total protein membrane images are presented in Supplementary Material Fig. S2. The molecular weight marker (HiMark pre-stained protein standard, ThermoFischer Scientific) was loaded in lane 1 (mm) and sizes are indicated on the right-hand side of each panel. Samples derived from the same experiment were blotted for VDAC and ANT (2 separate membranes due to similar size), and samples derived from the same experimental procedure, but on a different day for ATM, are shown here.

This was confirmed with western blotting that utilized antibodies against ATM protein kinase, VDAC and ANT 1/2/3/4 (IMM marker) as shown in Fig. 3. Digitonin permeabilisation was stopped at 0 min, 15 min, 20 min and 30 min time points. The cellular membrane and nuclear (CN), cytosolic (C), and M/S-isolated mitochondrial (M) fractions were included and compared to the separated OMM and mitoplast (IMM) fractions. ATM protein kinase was consistently associated with the mitoplast fraction, and not the OMM fraction. Further sub-fractionation of the mitoplast fractions showed that ATM is associated with the IMM fraction of the mitoplast and not the matrix (Supplementary Material Fig. S3).

### ATM plays a functional role in Complex I-mediated cardiac oxidative phosphorylation

A small but significant reduction in mitochondrial respiration rates has previously been reported in ATM-deficient lymphoblastoid cells with a rezasurin assay^20^. In light of the observed consistent association between ATM and the IMM of cardiac mitochondria, we investigated the effect of acute ATM inhibition on oxidative phosphorylation in isolated hearts obtained from 12-week old male Wistar rats (n = 10; 295.7 ± 17.45 g). The hearts were stabilised and subjected to retrograde Langendorff perfusion with either DMSO (0.03%, dimethyl sulphoxide, n = 5) or 3 µM KU60019 (ATM-specific inhibitor dissolved in DMSO, n = 5) for 20 minutes prior to mitochondrial isolation in KE buffer. This concentration of the inhibitor does not influence isolated cardiomyocyte cell viability (Supplementary Material Fig. S4), and was consequently used for whole heart retrograde perfusion prior to mitochondrial isolation. ATM protein levels in isolated cardiomyocytes were also determined in the presence and absence of KU60019 with western blotting (Supplementary Material Fig. S5).

Mitochondrial oxygen uptake was measured with a Clarke-type electrode (HansaTech.) and State 2 (basal oxygen consumption), State 3 (active respiration in presence of ADP), and State 4 (resting respiration after ADP usage) were recorded respectively in glutamate (5 mM) + malate (2 mM; GM) and palmitoyl-L-carnitine (0.45 mM) + malate (2 mM; PCM) respiratory buffers. Respiratory control ratio (RCR, State 3 divided by State 4) reflects the control of oxygen consumption by phosphorylation and did not differ significantly between control and KU60019 treated hearts. The substrates glutamate and malate, investigate the mitochondrial aspartate shuttle that produce NADH which is oxidized by complex I (NADH dehydrogenase) of the electron transfer chain (ETC) to NAD^+^^29^. Oxidation of NADH donates an electron to complex I which is used for ATP production. The ADP/O ratio indicates the number of ADP molecules added for each oxygen atom consumed and is an index of the efficiency of oxidative phosphorylation. This ratio differed significantly (p = 0.0298) for the complex I-mediated reduction of the NADH-linked substrates, GM, in KU60019-perfused hearts compared to controls. Together with a reduction in complex I respiratory rate, this resulted in a significant reduction (p = 0.0024) in the oxidative phosphorylation rate (ADP/min/mg mitochondrial protein) in GM, but not PCM respiratory buffer.

### Inhibition of ATM decreases enzyme-bound NAD(P)H excited state fluorescence lifetime

Rat cardiomyoblast cells (H9c2 cells) were treated with either DMSO (vehicle; n = 3) or 3 μM KU60019 dissolved in DMSO (n = 3) for 3–6 hours prior to FLIM. Fluorescence decays obtained at each pixel were fitted with a two-component model that yielded good fits (χ^2^ values = 1.041 ± 0.05; 1.045 ± 0.044; mean values ± s.d.). Fluorescence decay parameters were recorded for τ-_bound_, α-_bound_ (Fig. 5) and τ_-free_ (Supplementary Material Fig. S6). In line with previous reports that have found that τ-_bound_ varies with changes in metabolism^30–32^, we found that KU60019 treatment significantly decreased τ-_bound_ mean values (±s.d.) to 2.57 ± 0.14 ns (n = 3) compared to DMSO-treated cells (2.77 ± 0.26 ns; p = 0.02, Student’s two-tailed t-test), whereas α-_bound_ increased, albeit not significantly (p = 0.14). Similarly, inhibition of ATM with KU60019 in HEK293 cells, significantly reduced τ_-bound_ mean values (±s.d.) to 2.66 ± 0.11 ns compared to the control treated HEK293 cells (2.99 ± 0.11 ns; p = 0.0031) (Supplementary Material Fig. S7). No difference was observed in α-_bound_ values, although a significant reduction was observed in KU60019-treated HEK293 cells compared to control-treated HEK293 cells in τ_-free_ values (p = 0.0474, Supplementary Material Fig. S8).

## Discussion

This study shows that ATM protein kinase is consistently associated with the IMM of cardiac mitochondria isolated from 12-week old male Wistar rats, and shows that the inhibition of ATM affects complex I-mediated oxidative phosphorylation. This was mediated by an increased number of oxygen atoms consumed for each ADP molecule added (ADP/O ratio) that consequently decreased the mitochondrial coupling efficiency, and resulted in reduced oxidative phosphorylation and ATP production. Moreover, the inhibition of ATM resulted in a significant decrease in NAD(P)H fluorescence decay lifetime which suggests an increase in NADH and supports the notion that complex I-mediated NADH oxidation is impaired by the inhibition of ATM. Likewise, inhibition of complex I-mediated oxidative phosphorylation with rotenone results in a decrease in NAD(P)H fluorescence and have been associated with an increase in NADH relative to NADPH, potentially suggesting metabolic disturbance^33^.

Similarly to Morita *et al*.^22^, we made use of detergent-based subcellular fractionation of rat cardiac mitochondria to obtain mitoplasts. ATM was found to be consistently associated with the IMM and expands on previously published work that shows that the absence of ATM is associated with mitochondrial dysfunction in A-T patient derived fibroblasts^20^ and ATM^−/−^ mice thymocytes^21^. However, this is, to the best of our knowledge, the first evidence to show that ATM is located on the inner mitochondrial membrane of rat cardiac mitochondria. The removal of the OMM and integrity of the mitoplasts were confirmed with TEM (Fig. 1), and showed that 1.2% digitonin permeabilisation for 20 minutes was sufficient to remove the OMM completely whilst not rupturing the mitoplasts. Mitoplast morphology was compared with other published protocols, and showed similarity in morphology to mitoplasts obtained from rat liver mitochondria isolated with mechanical methods, including sonication^34^ and mitoplasts obtained with digitonin-permeabilisation^35^. The OMM marker, VDAC is attached at contact points to the IMM and insufficient uncoupling during separation results in traces of the protein being visible in the mitoplast fraction (Fig. 3). OMM contamination, due to contact points with the IMM, is often seen in studies that make use of osmotic lysis to obtain mitoplasts^36,37^ for mitochondrial patch-clamping experiments. The use of digitonin however, is known to decrease the presence of VDAC over time^38^, and to ensure this was the case in our study we systematically tested four time points to determine at what time point VDAC as an outer membrane marker and contact point contamination was no longer present, whilst the inner membrane marker (ANT) remained intact. At 20 minutes the majority of mitoplasts remained intact (as observed with TEM, Fig. 1e,f). Interestingly, very little TOM20 (Supplementary Material Fig. S1) is seen after 20 min 1.2% digitonin permeabilisation, and confirms complete removal of the outer membrane. However, irrespective of VDAC association with the remnant OMM observed in the IMM fraction, the outer mitochondrial membrane fraction did not contain any observable ATM protein kinase and no observable ANT 1/2/3/4, except at the initial time point directly after the addition of digitonin (Figs 2b,c and 3 OMM), at which time the mitochondria have not yet been permeabilised by digitonin. This time point served as a positive control for the outer mitochondrial membrane and mitoplast fraction, prior to permeabilisation. Although SR-SIM could confirm that ATM is not associated with the OMM whilst being present in the mitoplast fraction, it is not necessarily exclusively located on the inner membrane. This study shows also the effective use of SR-SIM as an imaging technique for isolated mitochondria and labelled proteins, however discernible mitoplast morphology is lost after 20 minutes due to permeabilisation of the outer membrane proteins, VDAC (Fig. 2) and TOM20 (Supplementary Material Fig. S1), and the absence of a labelled IMM marker that is not influenced by the loss of the OMM. ATM could not be used to distinguish mitoplast structure with SR-SIM after 20 minutes digitonin treatment due to punctate formation, and suggests that the protein could be facing towards the intermitochondrial space between the inner and outer membrane which would expose it to partial digitonin treatment. It is clear that greater resolution and more sensitive microscopy techniques, such as super resolution correlative light and electron microscopy (CLEM) are required to resolve the inversion of the mitoplast with sonication.

**Figure 4:**
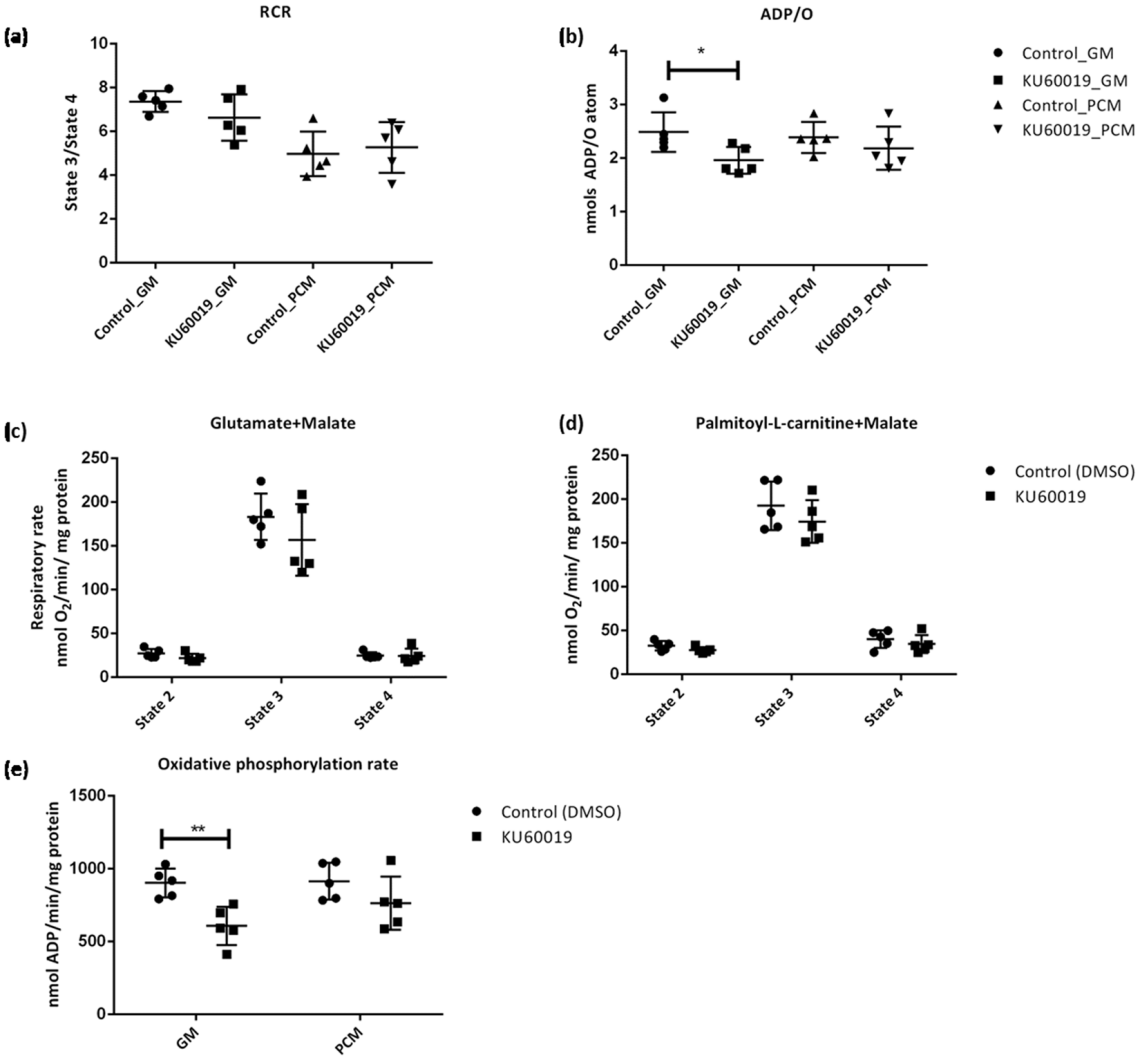
Inhibition of ATM decrease complex I mediated oxidative phosphorylation rate due to decreased oxidative phosphorylation efficiency and respiratory rate. The ATM inhibitor, KU60019, did not affect (**a**) the respiratory control ratio (State 3/State 4) but significantly decreased the (**b**) oxidative efficiency (ADP/O) of the ETC in complex I-mediated glutamate/malate (GM) respiration (p = 0.0298; Student’s t-test) but not in palmitoyl-L-carnitine/malate (PCM) mediated β-oxidation. The inhibition of ATM did not reduce the respiration rate significantly in either (**c**) GM or (**d**) PCM substrates, but decreased (**e**) the oxidative phosphorylation rate significantly (p = 0.0024; 2-way ANOVA, Bonferroni post-hoc test). Values correspond to 10 individual experiments (0.03% DMSO control, n = 5; KU60019, n = 5), and error bars are presented as mean ± standard deviation.

The IMM consists of mitochondrial cristae and contains the electron transport complexes as well as ATP synthase which is responsible for energy conversion and ATP production^39^. The location of ATM protein kinase (Fig. 3) on this membrane implies a possible role in oxidative phosphorylation, and is supported by the observation that silencing of ATM in lymphoblastoid cells results in a decrease in oxidative respiration as well as decreased membrane potential^20^. Similarly, the current study shows that the inhibition of ATM results in a significant decrease in complex I-mediated oxidative phosphorylation (GM-substrate) by significantly lowering the oxidative phosphorylation efficiency (ADP/O ratio, Fig. 4b) which, together with a decrease in oxygen consumption (Fig. 4c), results in a significant decrease in the oxidative phosphorylation rate (Fig. 4e). Interestingly, inhibition did not decrease the RCR ratios (coupling efficiency), nor significantly decreased any of the measured parameters in a PCM substrate. Palmitoyl-L-carnitine is imported into the mitochondrial matrix where it undergoes β-oxidation and produce Co-enzyme A (CoA), NADH and FADH_2_ (flavin adenine dinucleotide). NADH transfers electrons to the ETC at complex I, whilst the FADH_2_ transfers electrons to the ETC at complex II, and is the main source of ATP production in a healthy heart^18^. The glutamate-malate substrate assesses the aspartate-malate shuttle that produces NADH which provides electrons to complex I, and suggests that the inhibition of ATM has a larger effect on complex I mediated NADH oxidation than FADH_2_ oxidation at complex II. *Atm*-deficient mice thymocytes also exhibit a decrease in complex I enzyme activity and reduced ATP synthesis, but increased oxygen respiration which has been suggested to be context specific^21^. For example, heterozygous ATM^+/−^ mice shows reduced complex I activity in the liver, but not in the heart^5^. However, the latter study suggests that accumulating nuclear double strand breaks in the heterozygous model could lead to mitochondrial dysfunction^5^. The same research group found significantly increased markers of oxidative stress in ATM^+/−^ mouse hearts compared to wild-type litter mates, which was significantly reduced when fed a mitochondrial specific anti-oxidant^40^. Consequently, the underlying mechanism for ATM in this context still remains unknown, and warrants further research to disseminate between acute (post-transcriptional) and chronic responses where DNA damage could lead to mitochondrial dysfunction in response to the absence or inhibition of ATM.

The acute inhibition of ATM decrease Cytochrome C oxidase (COX, or complex IV) activity in permeabilised muscle tissue in a post-transcriptional manner (within an hour of KU55933 treatment), and is correlated with an increase in G6PD, which suggests a shift towards glycolysis^41^. ATM promotes glucose flux through the PPP by increasing both the abundance as well as activity of G6PD, consequently increasing NADPH production which is required for the anti-oxidant response in cells^11^. Moreover, the inhibition of COX increases ROS production and electron leak at complex I or III by reducing the redox centres of these complexes^42^. A reduction in the redox centre of Complex I should thus lead to decreased NADH oxidation if ATM inhibition impacts complex I.

**Figure 5:**
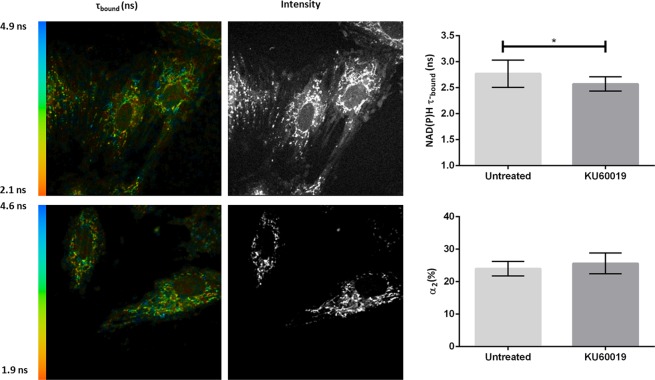
Inhibition of ATM decrease NAD(P)H fluorescence lifetime decay, but do not influence the percentage of enzyme-bound NAD(P)H. Representative fluorescence decay images of H9c2 cells treated with DMSO (vehicle control; top; n = 3) or KU60019 (3 μM; n = 3), which decreased NAD(P)H τ-_bound_ lifetime (ns) significantly (p = 0.02, Student’s t-test, mean ± s.d.), but did not influence α-_bound_.

To test this hypothesis we utilised two-photon fluorescence lifetime imaging microscopy (2P-FLIM) which is sensitive to microenvironment changes at the molecular level and can distinguish between free (τ1) and enzyme-bound NAD(P)H (τ2) based on fluorescence lifetime (where τ1 > τ2), which makes it a useful tool for probing metabolism in living cells^32^. Reduced NADH, and its phosphorylated form, NADPH are autofluorescent electron donors with identical fluorescent spectra, and is referred to as NAD(P)H due to the uncertain origin of the signal^33^. Changes in the lifetime of NAD(P)H are determined by fitting an exponential function to the observed time-resolved fluorescence decay, and have been used to distinguish between mitochondrial protein localisation^43,44^, redox ratio^30,45^ and glucose carbon diversion^32^. It has been suggested that the protein-bound form of NADH localizes mainly within the mitochondria whilst free NADH is primarily located within the cytosol^30,46^. We observed a bound lifetime (τ-_bound_) of 2.76 ± 0.26 ns (Fig. 5), in agreement with previously reported mean values (±s.d.) of 2.8 ± 0.2 ns in isolated liver mitochondria^46^, 2.7 ± 0.2 ns in HEK293 cells^33^, and 2.5 ns in isolated mice cardiomyocytes^25^, whilst inhibition with KU60019 resulted in a significant reduction of NAD(P)H fluorescence lifetime decay to 2.57 ± 0.14 ns (Fig. 5). This finding was confirmed in HEK293 cells (Supplementary Material Fig. S7). Inhibition of ETC respiration in cardiomyocytes with cyanide resulted in a similar significant decrease in NAD(P)H lifetime and caused an accumulation of NADH, which suggests that the NADPH/NADH balance defines the enzyme bound NAD(P)H lifetime^25^. Inhibition of complex I with rotenone in HEK293 cells resulted in a significant decrease of τ-_bound_ from 2.7 ± 0.2 ns to 2.52 ± 0.05 ns in both the cytosol and mitochondria of the cells, and suggests that the inhibition of complex I, and thus inhibition of NADH oxidation, can increase the concentration of NADH present in the cell relative to the NADPH concentration^33^. Taken together, the observed decreased oxidative phosphorylation rate and decreased NADH oxidation, suggests that ATM plays a functional role in the ETC and oxidative phosphorylation. No significant changes were observed in either α-_bound_ or τ_-free_, in line with previous reports that NAD(P)H τ2 can effectively and non-invasively quantify diversions of carbon away from the TCA cycle or the ETC^32^ (Supplementary Material Fig. S6,). The observed decrease in cellular NAD(P)H in the presence of the ATM inhibitor, KU60019, in this study agrees with the observation that cells expressing the C2291L-ATM mutant construct has a decreased flux of glucose through the PPP, consequently increasing glucose consumption and lactate generation through glycolysis and accordingly decreasing NADPH^11^. In light of these results, we suggest that the absence or inhibition of ATM does not only result in metabolic disturbances, but also mitochondrial dysfunction. This can be either through a decrease in NADPH because of an increase in NADH, which compromises the mitochondrial ROS scavenging systems or by shifting the cell metabolism towards glycolysis, which is often associated with pathological conditions in the heart. ATM knock-out mouse models develop hypertrophy and fibrosis, and it has been shown that ATM is required for the normal activation of the cardiomyocyte survival pathways^6^. Substrate selection is highly relevant in heart pathology^47^ and shifts from free fatty acid oxidation and decreased oxidative phosphorylation, as well as increased glycolysis is seen in hypertrophied hearts^48^ and in progressive heart failure^49^.

We showed that ATM is located within cardiac mitochondria under normoxic conditions, and our results contribute towards a better understanding of the potential role of ATM in mitochondrial function through ETC complex I, as well as its potential contribution towards the development of cardiovascular dysfunction by decreasing oxidative phosphorylation efficiency. The results presented here stress the need to further establish the role of ATM and determine whether ATM directly phosphorylates mitochondrial proteins, is activated in response to mitochondrial damage^50^ and can be imported in response to stress. Our results indicate all are possible.

## Materials and Methods

All the procedures involving animals were in compliance with the South African National Standard for the care and use of animals for scientific purposes (SANS 10386:2008); the Medical Research Council Guidelines on Ethics for Medical Research; Book 3: Use of Animals in Research and Training and the National Institutes of Health Office of Laboratory Welfare: The Guide for the Care and Use of Laboratory Animals, 8th Edition; USA, and was approved by the Research Ethics Committee: Animal care and Use, Stellenbosch University, South Africa (approval numbers SU-ACUM12-0040; SU-ACUD16-00179).

### Mitochondrial isolation for subfractionation and oxidative phosphorylation analysis

Male Wistar rats were injected with Eutha-naze (Bayer, 200 mg/ml at an overdose of 200 mg/kg via intraperitoneal injection performed by a registered animal laboratory technologist, South African Veterinary Council, SAVC number: AL16/15486), and euthanised through exsanguination after rapid excision of the heart.

Mitochondrial isolation was performed with a standard differential centrifugation protocol^51^. For oxidative active mitochondria, all isolations were performed in ice-old mitochondrial isolation buffer (0.018 M KCl, 0.01 M EDTA, pH 7.4). Briefly, the ventricular tissue was homogenised with a Heidolph SilentCrusher M, followed by a slow centrifugation step (755 × g for 10 minutes at 4° Celsius) in order to obtain a pellet containing the nuclei, cell membranes and remaining tissue. The supernatant was transferred to a clean centrifuge tube and centrifuged at 18 800 × *g* for 10 minutes at 4 °C to obtain a mitochondrial pellet. The pellet was re-suspended in ice-cold mitochondrial isolation buffer with a 2 cm^3^ Potter-Elvehjem glass-teflon homogenizer, and stored on ice until use.

For subfractionation, the atria were removed directly after excision, and the remaining ventricular tissue was placed in ice-cold D-mannitol/sucrose buffer adapted from^52^ and^53^ (M/S buffer; 70 mM sucrose, 220 mM D-mannitol, 10 mM HEPES, 0.5 M EGTA, pH to 7.2 with KOH), finely diced, and subjected to homogenization (Heidolph SilentCrusher M). Mitochondria were isolated as described above and resuspended in ice cold M/S buffer. The mitochondrial fraction was washed with centrifugation at 10 000 × *g* for 10 min at 4 °C, and resuspended in ice-cold M/S buffer. Protein concentration was determined with a Bradford protein assay^54^ prior to 1.2% digitonin treatment (Sigma, D141). A volume equal to 100 μg mitochondria was aliquoted into a clean Eppendorf tube, and an equal volume of 1.2% digitonin (prepared by boiling for 1 minute in M/S buffer) was added. An aliquot was taken at the moment of addition (0 minute time point), and the reaction was quenched in a 10x dilution with ice-cold M/S buffer containing bovine serum albumin (BSA, 25 μg/ml). The master digitonin-permeabilisation reaction was allowed to continue for 30 min whilst gently stirring at 0° Celsius, and aliquots were taken at 15 min, 20 min and 30 min time points, and quenched as previously described. The quenched 10x dilution time-point reactions were subsequently centrifuged at 10 000 × *g* for 10 min at 4 °C (Sigma 1–14 K) to obtain the mitoplasts (sediment), and the supernatant was carefully aspirated. The supernatant was centrifuged at 100 000 × *g* for 1 hour at 4° Celsius to obtain the OMM fraction.

### Transmission electron microscopy and super-resolution structured illumination microscopy

In brief, samples were processed with a Leica EM tissue processer and were scoped with a JEOL 10–11 transmission microscope (JEOL,Japan) and a SIS imaging according to standard protocol (National Health Laboratories Services, Tygerberg Hospital, Cape Town, South Africa).

Super-Resolution Structured Illumination Microscopy (SR-SIM) was performed at the Stellenbosch University Central Analytical facility (CAF) Fluorescence Microscopy unit. Mitochondria and mitoplasts were prepared as described previously and fixed with 2.5% glutaraldehyde. The samples were incubated overnight with either ATM (Abcam, anti-rabbit, mAb, ab199726^55,56^) and VDAC (CST, anti-mouse), or ATM and TOM20 (Abcam, anti-mouse), washed and probed with the appropriate fluorescently Goat Anti-Rabbit IgG H&L (Alexa Fluor® 488, ab150077) and Goat Anti-Mouse IgG H&L (Alexa Fluor® 594, ab150116) secondary antibody.

Samples were acquired with a Zeiss LSM 780 ELYRA PS1 microscope. Thin (0.1 μm) z- stacks of high-resolution image frames were collected with 5 rotations by using an alpha Plan-Apochromat 1006/1.46 oil DIC M27 ELYRA objective and an ELYRA PS.1 (Carl Zeiss Microimaging) microscope equipped with a 488 nm laser (100 mW), 561 nm laser (100 mW) and Andor EM-CCD camera (iXon DU 885). Micrographs were reconstructed using ZEN software (black edition, 2011, version 7.04.287) using a structured illumination algorithm^57^.

### Langendorff perfusions (for mitochondrial analysis)

Excised hearts (n = 10) were cannulated via the aorta and perfused with Krebs-Henseleit buffer (KHB; 119 mM NaCl, 24.9 mM NaHCO_3_, 4.7 mM KCl, 1.2 mM KH_2_PO_4_, 0.6 mM MgSO_4_·7H_2_O, 0.59 mM Na_2_SO_4_, 1.25 mM CaCl_2_·12H_2_O and 10 mM glucose; pH 7.4) at a constant pressure of 100 cm H_2_O and gassed with 95% oxygen and 5% carbon dioxide. Hearts were stabilized for 20 minutes, after which they were perfused in a recirculating manner with KHB containing 3 μM KU60019 (n = 5) or an equal volume DMSO (0.03%; n = 5). Protein determination for the mitochondrial data was performed with a Lowry assay^58^.

### Oxygen consumption in isolated mitochondria

Mitochondrial oxygen consumption was analysed with a Clarke-type oxygraph (Oxytherm system, Hansatech Instruments) immediately following isolation. Briefly, two chambers containing a glutamate or palmitoyl-L-carnitine assay buffer (250 mM sucrose, 10 mM TRIS-HCl (pH7.4), 8.5 mM K_2_HPO_4_-3H_2_O, 5 mM malate and 5 mM glutamate or 0.45 mM palmitoyl-L-carnitine; pH 7.4), equilibrated to ambient oxygen levels at 25 °C, were analysed in parallel. Data was captured polarographically with HansaTechPlus software. Oxygen consumption prior to the addition of ADP (State 2), in the presence of ADP (State 3), and after complete utilization of ADP (State 4) was measured as well as State 3 oxygen consumption after 20 minutes anoxia followed by reoxygenation was noted. These values were used to calculate the oxygen consumption (nmol O_2_/min/mg mitochondrial protein), oxidative phosphorylation rate (nmol ADP/min/mg mitochondrial protein), respiratory control rate (RCR, State 3/state 4), and the ADP/O ratio.

### Western blotting

Briefly, the mitochondrial, mitoplast, OMM, IMM and matrix fraction lysates were prepared for western blotting with the addition of one volume M/S buffer and one volume 1x SDS buffer (10% Glycerol, 50 mM TRIS-HCl, pH 6.8, 30 mM β-glycerophosphate, 1 mM NaVO_4_, 0.5 mM NaF, 2% SDS). The samples were boiled for four minutes and stored at −80° Celsius until use. Samples were boiled again for 4 minutes prior to being loaded on 4–15% Mini-PROTEAN^®^ TGX™ Stain free Precast Protein Gels (Bio-Rad Laboratories), and subjected to electrophoresis for 1 hour. Due to major size differences in the proteins investigated (ATM protein kinase #2873, 350 kDa, Cell Signaling Technology (CST)^59^; VDAC #4866, 32 kDa, CST; and ANT 1/2/3/4 #H-188, 34 kDa Santa Crus Biotechnology^60^), a modified transfer buffer was used (25 mM TRIS, 190 mM glycine and 10% methanol v/v), and the gels were allowed to rest for 30 minutes in the buffer prior to activation in the ChemiDoc MP system (Bio-Rad Laboratories), and transfer. Transfer was performed at 200 V and 200 mA for 1 hour 30 minutes at 4° Celsius onto an Immobilon®-P PVDF membrane (EMD Millipore). The membranes were briefly placed in MeOH, and allowed to dry before blocking in a 5% liquid fat-free milk solution. The membranes were cut below the 170 kDa molecular mark for ATM, and probed from 170 kDa to- 450 kDA for ATM. For VDAC and ANT1/2/3/4 the membranes were cut just below the 55 kDa and probed from approximately 20 kDA to just above 43 kDA. The antibodies were diluted 1:1000 as follows: SignalBoost™ Immunoreaction Enhancer Kit (EMD Millipore) for ATM protein kinase antibody (CST); TBS-T for VDAC antibody and secondary antibody, and TBS-T (20 mM Tris-HCl (pH 7.6), 137 mM NaCl, 0.1% Tween-20) with 2.5% fat free milk solution for ANT 1/2/3/4 (SCBT) and its secondary antibody. Donkey anti-rabbit secondary antibody (HRP-linked secondary antibody #7074, CST) was diluted 1:4000 in either SignalBoost™ Immunoreaction Enhancer Kit (EMD Millipore) for ATM detection, TBS-T for VDAC and TBS-T + 2.5% milk for ANT1/2/3/4. After primary and secondary antibody incubation the membranes were treated with Clarity™ Western ECL Blotting Substrate and visualised on the ChemiDoc MP system on the Chemi Hi sensitivity setting, which was stopped prior to overexposure (which appears red on program settings).

### Cell culture

H9c2 (2-1) cells were obtained from ATCC® (CRL-1446™, passage 14) and maintained in DMEM (Gibco) with 4 mM L-Glutamine, 4.5 g/L glucose, 10% FBS and 1% penicillin/streptomycin at 37 °C and 5% CO_2_. Cells were seeded at a concentration of 2.4 × 10^4^ cells/ml on 35 mm MaTIK plates 48 hours prior to treatment, and treated with either DMSO (0.05%, n = 3) or 3 μM KU60019 (n = 3), 3–6 hours prior to imaging. HEK293 cells were previously obtained from ATCC (CRL-1573™), and were maintained in MEM (Gibco) with 2 mM-L-Glutamine, 10% FBS and 1% pencillin/streptomycin at 37 °C and 5% CO_2_. Cells were seeded at a concentration of 1.5 × 10^5^ cells/ml on 35 mm MaTIK plates for 48 hours prioris to treatment. The HEK293 cell cultures were treated identical to the H9c2-cell cultures (0.05% DMSO, n = 3 or 3 μM KU60019, n = 3).

### Fluorescence lifetime imaging microscopy

These experiments were performed at the Rutherford Appleton Laboratory, the Science and Technology Facility Council, Oxfordshire, UK. Fluorescence intensity and lifetime images were acquired using a custom-built multiphoton fluorescence lifetime system around a modified inverted microscope (TiE, Nikon). Samples were imaged using a 60× , NA 1.2 water-immersion objective^61,62^. Multiphoton excitation was generated from a titanium: sapphire laser, tuned to 760 nm, operating at 76 MHz with 200 fs pulse width (Coherent, USA). A BG39 filter was used to block the near infra-red light before non-descan detection using a hybrid detector, HPM-100 (Becker and Hickl, GmbH). A pixel dwell time of 5.04 μs was used for 256 × 256 pixel images over a 212 μm × 212 μm field of view. TCSPC electronics (SPC 830, Becker & Hickl, Berlin, Germany) were used to acquire fluorescence decay curves with 256 time bins. Following a 3 × 3 binning of photons and a threshold of 25, two-component fluorescence decay curves were used to fit the decay curves for FLIM images using iterative reconvolution in SPCImage (Version 7.1, Becker and Hickl)^33^. NAD(P)H fluorescence lifetime parameters were obtained from the FLIM data and reported as an average of 10 pixels over at least three to five regions of three separate cultures imaged on different days.

### Statistical analysis

The data were analysed using GraphPad Prism 6.0 software (GraphPad Software, Inc., La Jolla, CA). Statistical comparisons were made using a two-tailed Student’s t-test or 2-way analysis of variance (ANOVA) followed by a Bonferroni post-hoc test where applicable. All values and error bars are presented as mean ± s.d, and the level of significance was set at p < 0.05.

## Supplementary information

Supplementary Information: Ataxia-Telangiectasia Mutated is located in cardiac mitochondria and impacts oxidative phosphorylation

## Data Availability

The datasets generated and analysed during the current study are available from the corresponding author on request.
