# Supplementary material for: Ataxia-Telangiectasia Mutated is located in cardiac mitochondria and impacts oxidative phosphorylation
Source: Sci Rep. 2019 Mar 18;9:4782. doi: 10.1038/s41598-019-41108-1 (PMC6423017; doi:10.1038/s41598-019-41108-1)
Supplement: Supplementary file 1 — Supplementary Information: Ataxia-Telangiectasia Mutated is located in cardiac mitochondria and impacts oxidative phosphorylation [file 41598_2019_41108_MOESM1_ESM.pdf]

Supplementary Information:

Title: Ataxia-Telangiectasia Mutated is located in cardiac mitochondria and impacts oxidative phosphorylation

Authors:

Marguerite Blignaut[1], Ben Loos[2], Stanley W. Botchway[4,5], Anthony W. Parker[4,6], Barbara Huisamen[1,3]

Departments where work was performed:

Department of Biomedical Sciences, Division Medical Physiology, Faculty of Medicine and Health Sciences, Tygerberg Campus, South Africa

Department of Physiological Sciences, Faculty of Sciences, Stellenbosch University, South Africa

Rutherford Appleton Laboratory, Science and Technology Facility Council, Oxfordshire, UK

Authors affiliations:

[1]Division of Medical Physiology, Department of Biomedical Sciences, Faculty of Medicine and Health Sciences, Stellenbosch University, Tygerberg, 7505.

[2] Department of Physiological Sciences, Faculty of Sciences, Stellenbosch University, South Africa

[3] Biomedical, Research and Innovation Platform, South African Medical Research Council, Tygerberg, 7505.

[4] Central Laser Facility, Research Complex at Harwell, STFC Rutherford Appleton Laboratory, Harwell Campus, Didcot, OX11 0QX, UK

[5] Oxford Brookes University, Department of Biological and Medical Sciences, Oxford, OX3 0BP, UK

[6] Department of Physics, Faculty of Science, Stellenbosch University, Private Bag X1, Matieland, South Africa, 7602.

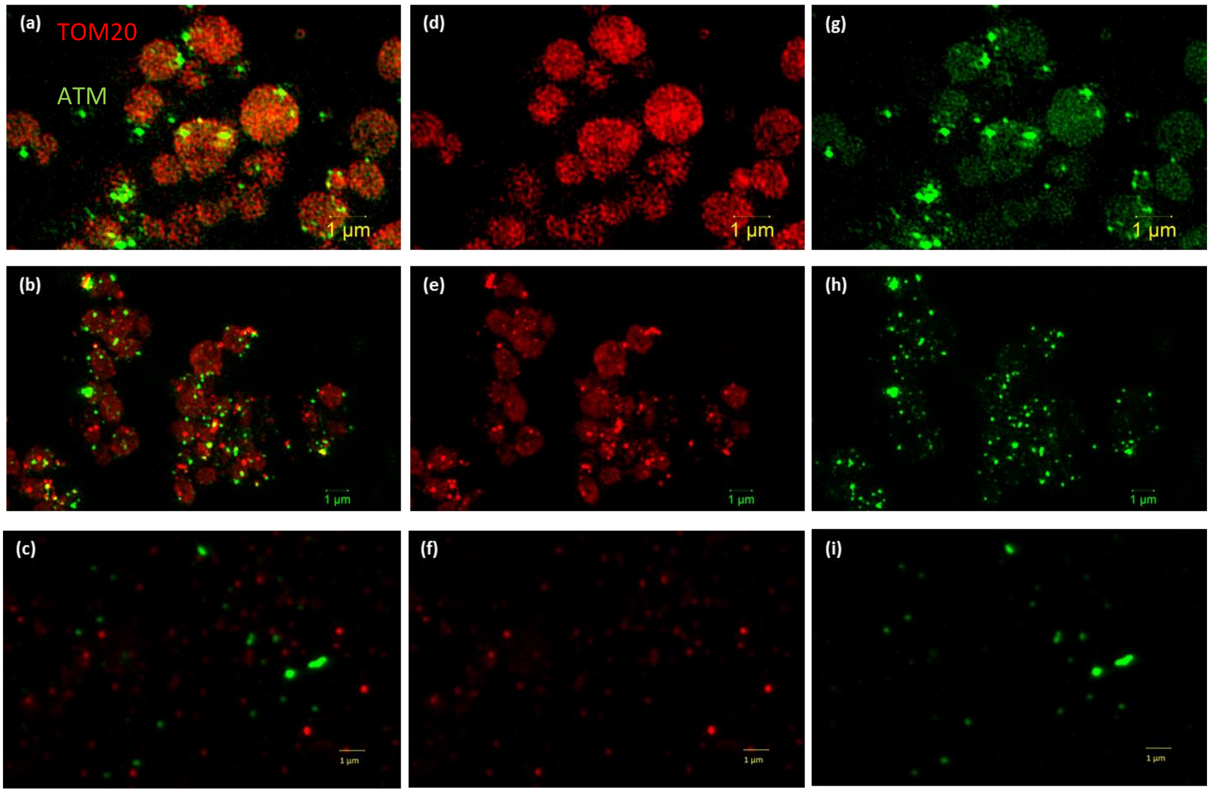

Supplementary Figure S1: **Super resolution structured illumination microscopy of isolated mitochondria and mitoplast preparation**. MS-isolated mitochondria (a, overlay) was probed with TOM20 (d; red) and ATM (g; green). Fig S1b (overlay of Fig. S1 e and h) shows the permeabilisation of the outer membrane (e; red) and formation of mitoplasts directly after the addition of 1.2% digitonin. Fig. S1c (overlay of Fig. S1f and i) represents the formation of mitoplasts after 20 minutes of digitonin digestion. Scale bars represent 1 µm.

Western blotting: Isolated proteins were separated either with hand-cast denaturing 7.5% acrylamide SDS-gels or 4–15% Mini-PROTEAN® TGX™ Precast Protein Gel (BioRad, #4561086) in a Mini-Protean vertical electrophoresis cell (BioRad), and transferred in a Mini-Transblot system (BioRad). All gels were imaged prior to transfer, and membranes were imaged after transfer with ChemiDoc MP System (BioRad) with size settings applicable to Mini-Protean gels. Membranes were cut to accommodate the different protein sizes (ATM, 350 kDa; VDAC (32 kDa) and ANT1/2/3/4 (34 kDa), where ANT1/2/3/4 and VDAC were probed on different membranes that were obtained from gels on which the same samples were separated in the same electrophoresis cell, and transferred in the same system. Images were saved as Image lab images, and exported from ImageLab V.5.0 (BioRad) software for publication without any adjustments.

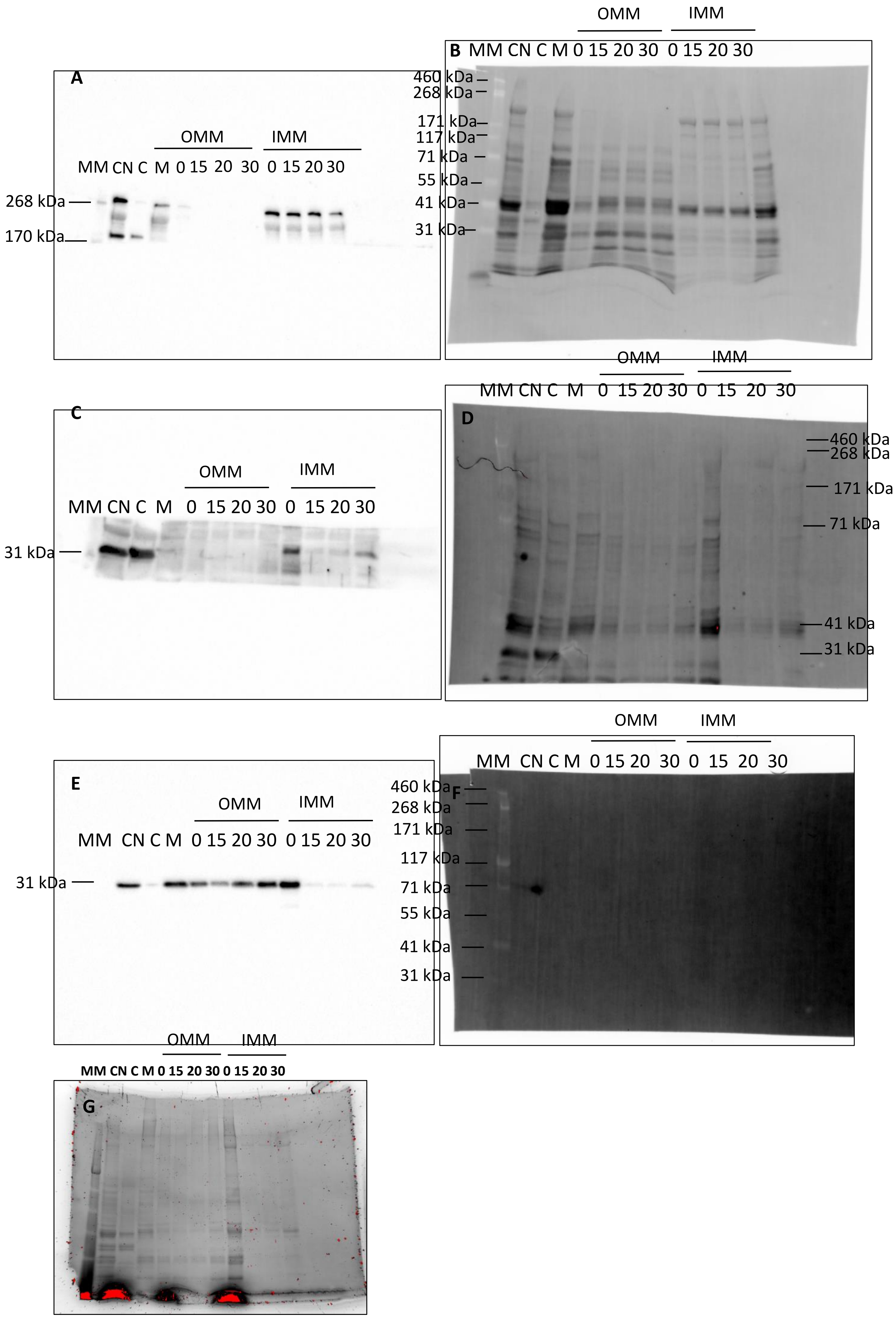
A
OMM
IMM
MM CN C M 0 15 20 30 0 15 20 30
268 kDa
170 kDa
B
OMM
IMM
MM CN C M 0 15 20 30 0 15 20 30
460 kDa
268 kDa
171 kDa
117 kDa
71 kDa
55 kDa
41 kDa
31 kDa
C
OMM
IMM
MM CN C M 0 15 20 30 0 15 20 30
31 kDa
D
OMM
IMM
MM CN C M 0 15 20 30 0 15 20 30
460 kDa
268 kDa
171 kDa
71 kDa
41 kDa
31 kDa
E
OMM
IMM
MM CN C M 0 15 20 30 0 15 20 30
31 kDa
F
OMM
IMM
MM CN C M 0 15 20 30 0 15 20 30
460 kDa
268 kDa
171 kDa
117 kDa
71 kDa
55 kDa
41 kDa
31 kDa
G
OMM
IMM
MM CN C M 0 15 20 30 0 15 20 30

Supplementary Figure S2: Western blot of subcellular fractionation of the mitochondrial fraction (M) into OMM and IMM/mitoplast with 1.2% digitonin: The images shown here were directly exported from ImageLab 5.0 (BioRad) without any modification. Fig S2a: Uncropped ATM western blot image and its total protein membrane (Fig S2.b) prior to being cut below the 170 kDa mark (1st lane, left; HiMark™ Pre-stained Protein Standard, Invitrogen, ThermoFischer) and probed overnight with anti-rabbit monoclonal ATM antibody (Cell Signalling Technologies, D2E2, 2873) and have been cited in 112 publications) . Fig S2.c: **Uncropped ANT 1/2/3/4** (left) and its total protein membrane (Fig s2.d) prior to being cut above the 41 kDa mark (1st lane, left; HiMark™ Pre-stained Protein Standard, Invitrogen, ThermoFischer) and probed overnight with anti-rabbit polyclonal ANT1/2/3/4 antibody (H-188, sc-11433, Santa Cruz Biotechnoglogy). **Figure 2e: Uncropped VDAC** and its total protein membrane (Fig S2.f) prior to being cut at the 41 kDa mark (1st lane, left; HiMark™ Pre-stained Protein Standard, Invitrogen, ThermoFischer) and probed overnight with anti-rabbit monoclonal VDAC (Cell Signaling technologies, D73D12, cited in 65 publications). Due to the dark transfer the original 4–15% Mini-PROTEAN® TGX™ Precast Protein Gel (BioRad, #4561086) image is included (bottom). All the membranes were loaded as follows: HiMark™ Pre-stained Protein Standard, Invitrogen, ThermoFischer, Nucleus and cell membrane fraction (CN), cytosolic fraction(C), M/S isolated mitochondrial fraction (M), OMM fraction at 0 min, 15 min, 20 min and 30 min 1.2% digitonin digestion, IMM (mitoplast consisting of inner mitochondrial membrane and matrix) fraction at 0 min, 15 min, 20 min and 30 min 1.2% digitonin digestion. Chemiluminescent exposure was performed in the ChemiDoc MP system on the Chemi Hi sensitivity setting.

Mitoplast subfractionation and Proteinase K digestion: In order to confirm that ATM is located on the inner mitochondrial membrane and not in the matrix of the cardiac mitochondria, we isolated mitochondria in M/S buffer (as described previously) and further separated the inner mitochondrial membrane and matrix as described by Greenewalt et al[1]. Briefly, the mitoplast fraction (pellet), obtained after digitonin permeabilisation, was gently suspended in half the volume of M/S buffer containing 16 mg/ml Lubrol WX (Sigma). The suspension was incubated on ice for 15 minutes, followed by centrifugation at 100 000x*g* for 1 hour at 4° Celsius. The pellet (inner mitochondrial membrane) as well as the supernatant was retained (matrix proteins) for Western blotting purposes. A total of 25 µg M/S-isolated mitochondria was subjected to Proteinase K digestion (0.1 µg/ml) and digested in the presence of 0.1 % Triton-X for 1 minute, 3 minutes, 5 minutes and 10 minutes. The reactions were quenched with 5 mM phenylmethyl sulphonyl fluoride (PMSF) on ice.

ATM was not visible at 1 min even with Triton-X disruption, but was detected after 3 and 5 minutes of Proteinase K digestion and Triton-X disruption (Fig S3). This supports the notion that the protein is

located on the inner mitochondrial membrane. Zhang et al[2] demonstrated that ATM can be rapidly degraded by Proteinase K in both the presence or absence of Triton-X, and suggested that the rapid degradation (within 5 minutes) is because of ATM's location on the outer membrane of the peroxisome. We did not observe ATM after 1 minute of digestion but could observe ATM after 3 and 5 minutes of digestion, and supports our digitonin-based observations that ATM is located on the IMM. Both VDAC and ANT 1/2/3/4 stayed intact after 10 min of Proteinase K digestion and Triton-X disruption, and this phenomenon has been observed previously[3].

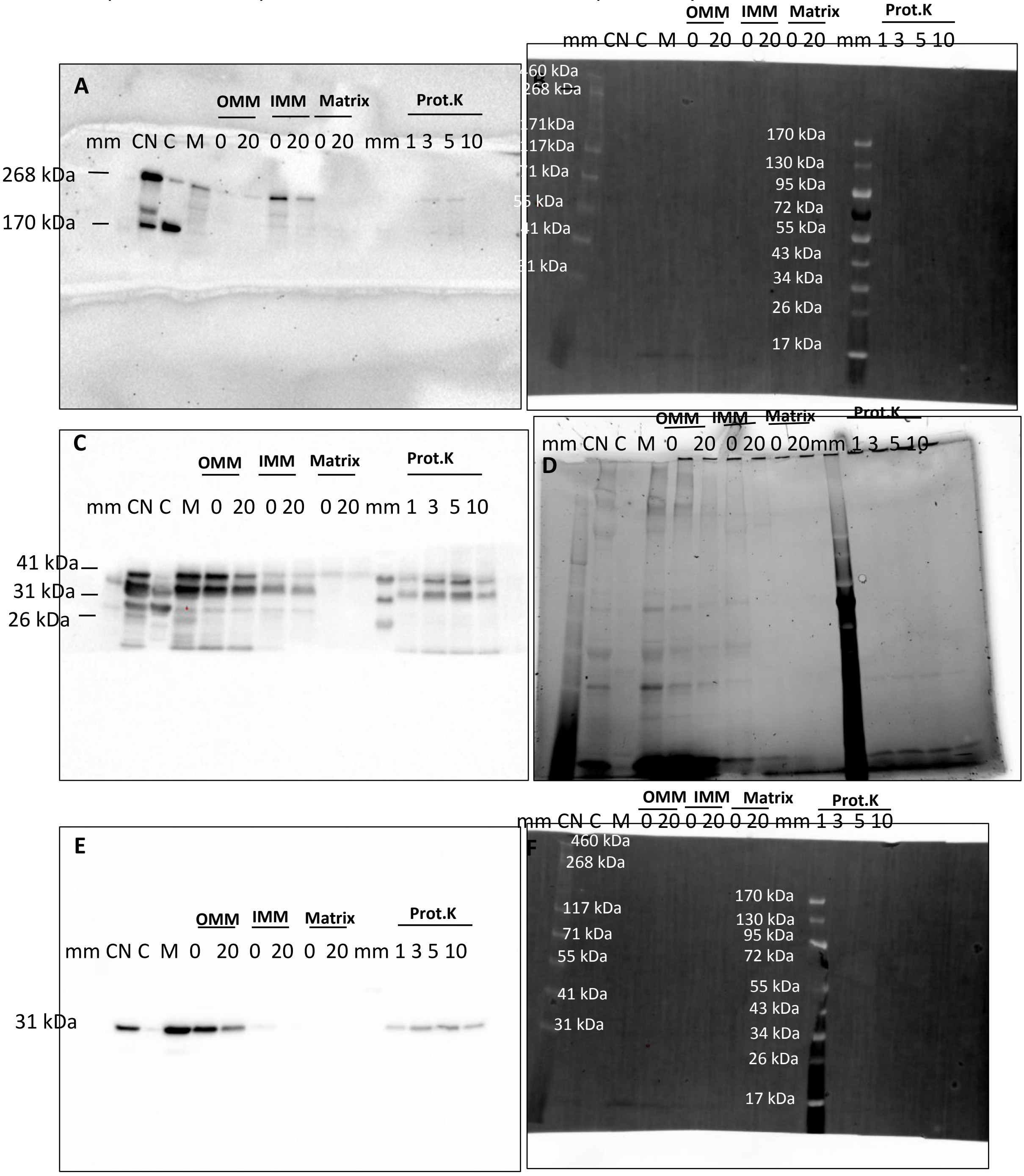

Supplementary Figure S3: Mitochondrial and mitoplast subfractionation and Proteinase K digestion: Isolated mitochondria was fractionated into outer mitochondrial (OMM) membrane and mitoplast fractions with 1.2% digitonin and the latter was further separated into inner mitochondrial membrane (IMM) and matrix fractions. The fractions were probed for ATM (Fig S3.a), ANT (Fig S3.c), same membrane as ATM, Fig S3.b) and VDAC (Fig S3.e). The total protein membranes are shown respectively in Fig S3.b (on which both ATM and ANT was probed), and Fig.S3.f. Figure S3d shows the corresponding 4–15% Mini-PROTEAN® TGX™ Precast Protein Gel (BioRad, #4561086) for Fig S3.b, which was loaded as follows: MM: HiMark™ Pre-stained Protein Standard, Invitrogen, ThermoFischer, Nucleus and cell membrane fraction(CN), cytosolic fraction (C), M/S isolated mitochondrial fraction (M), OMM fraction at 0 min and 20 min 1.2% digitonin digestion, IMM at 0 and 20 min 1.2% digitonin digestion, and the matrix fraction at 0 and 20 min 1.2% digitonin digestion; mm (PageRuler™ Prestained Protein Ladder, 10 to 180 kDa, Thermo Fischer Scientific), 1 min, 3 min, 5 min and 10 min M/S isolated mitochondrial Proteinase K digest fractions. The gels were loaded with the same lysates, electrophoresed in the same chamber and transferred in the same chamber. Due to incomplete transfer in the 41 kDa size region (Fig. S3.f), it is not possible to exclude the presence of VDAC in the matrix fractions.

Cardiomyocyte cell viability and ATM expression in cardiomyocytes: Prior to retrograde Langendorff perfusion of whole hearts with KU60019 for mitochondrial isolation, a cell viability assay was performed on cardiomyocytes isolated from young, male Wistar rats with DMSO (vehicle) and 3 µM KU60019 in DMSO to determine whether the ATM inhibitor influence cell membrane integrity and viability. ATM protein expression was determined with Western blot analysis in cardiomyocytes obtained from young, control male Wistar rats, and were either untreated, treated with 10 nM insulin, an activator of ATM phosphorylation[4], 100 nM wortmannin, a non-specific inhibitor of ATM[5], a combination of 10 nM insulin and 100 nM wortmannin, as well as 3 µM KU60019, and a combination of 10 nM insulin and 3 µM KU60019.

Briefly, ventricular cardiomyocytes were isolated from basal, control hearts (n = 3). Isolated cardiomyocytes were prepared essentially as previously reported (Fisher et al 1991). Male Wistar rats were anaesthetized with sodium pentobarbital (160 mg/kg), where after the hearts were rapidly removed and retrogradely perfused with a calcium free medium (HEPES buffer: 6 mM KCl, 1 mM Na_2HPO_4, 0.2 mM NaH_2PO_4, 1.4 mM $MgSO_4$, 128 mM NaCl, 10 mM HEPES, 5.5 mM glucose, 2 mM pyruvate, pH 7.4, 37 ^{o}C, equilibrated with oxygen) for 5 minutes. Thereafter, the perfusion was switched to a second medium (HEPES buffer containing 0.7% bovine serum albumin (BSA), fraction V, fatty acid free, 1.1 mg collagenase/mL and 15 mM 2,3-butanedione monoxime (BDM)). Following

15 minutes perfusion, the calcium concentration was raised in two steps to 200 uM over the next 10 minutes. Perfusion was continued to a total time of 35 minutes. The ventricles were carefully separated from the atria, minced with tweezers and suspended in the second medium given above but containing 1% BSA, 1% BSA fraction V, fatty acid free and half the concentration of collagenase and BDM. The suspension was placed in a cell culture flask in a shaking benchtop incubator (37 ^{o}C) and digested for a further 15 min, where after the calcium concentration was gradually raised to 1.25 mM over the next five minutes. The isolated cells were filtered through a nylon net (mesh size 200 x 200 um) and gently spun down (3 min., 100 rpm). The pellet obtained was resuspended in HEPES buffer containing 1.25 mM $CaCl_2$, 2% BSA (fraction V, fatty acid free) and the cells allowed to settle for 3-4 minutes. The supernatant was carefully aspirated, and the loose pellet resuspended in the same buffer and allowed to recover from the trauma of isolation for 1 hour before experimentation.

The viability (Fig S4) of the isolated cardiomyocytes routinely exceeded 80% as determined by the trypan blue exclusion method. The cells were untreated, treated with 0.03%, dimethyl sulphoxide, (DMSO) shown as vehicle or with 3 µM KU60019 dissolved in DMSO for 45 minutes in duplicate. Pellets, obtained by centrifugation, were re-suspended in fresh HEPES buffer prior to the addition of 5 µM PI. The samples were incubated in the dark at room temperature (~ 23°C) for 15 minutes before fluorescence was measured by FACS analysis. Twenty thousand events were acquired per condition.

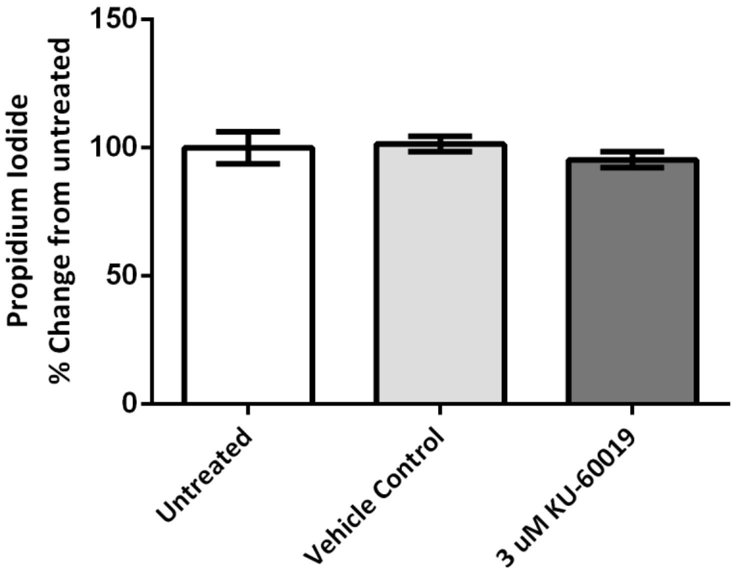

Supplementary Figure S4: Cell viability assay of isolated cardiomyocytes treated with 0.03% DMSO or 3 µM KU60019. Cell membrane integrity was maintained in the presence of 0.03% DMSO or 3 µM

KU60019 when treated with propidium iodide, and no change was observed between treated and untreated cardiomyocytes.

Total ATM protein expression levels were determined in isolated cardiomyocytes with Western blotting (Fig S5), and was normalised to the total protein membrane. The ATM inhibitor, KU60019 did not influence total ATM levels significantly, however, when combined with the known ATM activator, insulin, total ATM levels decreased significantly (p<0.0001) compared to ATM in cardiomyocytes treated with insulin alone.

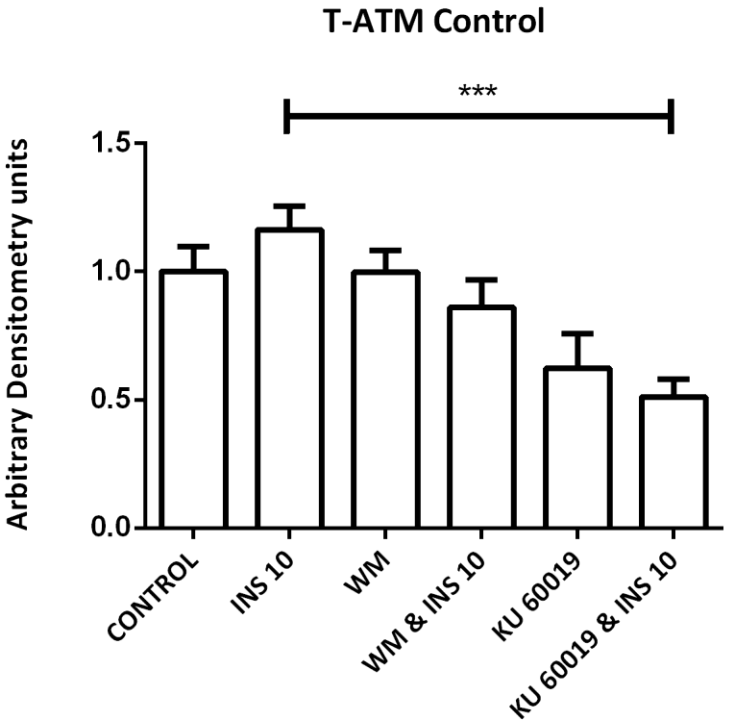

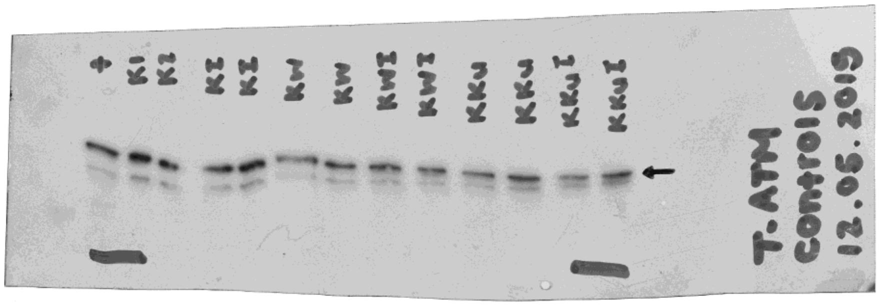

Figure S5: Western blot analysis of total- and phospho-ATM levels in isolated cardiomyocytes. Total ATM levels were determined with Western blotting in cardiomyocytes isolated from young, male Wistar rats. The blot shows a positive control (+) of total ventricular heart tissue, untreated controls (K), 10 nM insulin (KI), 100 nM wortmannin (KW), a combination of 10 nM insulin and 100

nM wortmannin (KWI), 3 µ KU60019 (KKu), and a combination of 3 µM KU60019 and 10 nM insulin (KkuI) treated cardiomyocyte lysates. n=4-8 per group analysed, One-way ANOVA, Bonferroni post-hoc test ****p<0.0001.).

Fluorescence lifetime imaging: Changes in autofluorescence decay lifetimes reflect changes in the bound NADPH/NADH ratio[6]. The balance between reduced NADH to oxidized NAD^+ reflects the balance of the TCA cycle which produces NADH to ETC activity that accepts the electron. Inhibition of the ETC will halt NADH oxidation, consequently resulting in ROS production[7]. On average, the lifetime decay of free (τ_1) NAD(P)H is considerably shorter than that of bound (τ_2), and it is thus possible to quantify the relative fractions of free (α_1) and bound (α_2) NADPH in a cell[8]. The above mentioned parameters are determined by fitting a time-resolved fluorescence decay curve[6,9,10] where τ_{bound} is reported as the weighted mean of the fluorescence lifetimes of the enzyme-bound species.

The relative fraction of free (α1) NAD(P)H decreased when treated with KU60019 (74.38±3.202) compared to control cells (76.00±2.259, Fig 4a), whereas the relative fraction of bound (α_2) NAD(P)H increased (control: 24.00±2.26; KU60019: 25.62±3.202; Fig4b), but these changes were negligible. The ratio of free and protein bound NAD(P)H (α_1/α_2) decreased, albeit not significantly (p=0.2, Fig 4c). No differences were observed in autofluorescence lifetime decay of freely diffusing NAD(P)H (τ_{free}; ns) between untreated and KU60019 treated cells, and is consistent with the suggestion that enzyme-bound NAD(P)H variation in τbound lifetime values seems to be specific to the type of metabolic disruption, such as mitochondrial dysfunction at Complex I[6], or the utilization of different substrates following inhibition of glycolysis [10].

The experiment was repeated in HEK293 cells with 0.03% DMSO (control) and compared to HEK293 cells treated with 3 µM KU60019 to determine if a similar decrease is observed in bound (τ2) lifetime decay (Fig S7). A significant decrease was observed in the τ-bound NAD(P)H values for KU60019 treated HEK239 cells (2.663 ±0.1090 ns) compared to the control treated cells (2.990 ± 0.1122 ns; p= 0.0031) . Similarly to the H9c2 cells, a significant reduction was observed in τ-bound NAD(P)H lifetime mean values (ns). The relative fractions of τ-bound NAD(P)H and τ-free NAD(P)H measured as α1(%) and α2(%) did not differ (Fig S8), which is similar to the observations made in H9c2 cells (Fig 5 and Fig S6). Interestingly, the τ-free NAD(P)H lifetime decreased significantly (p=0.0474) in HEK293 cells but not in H9c2 cells (Fig S8).

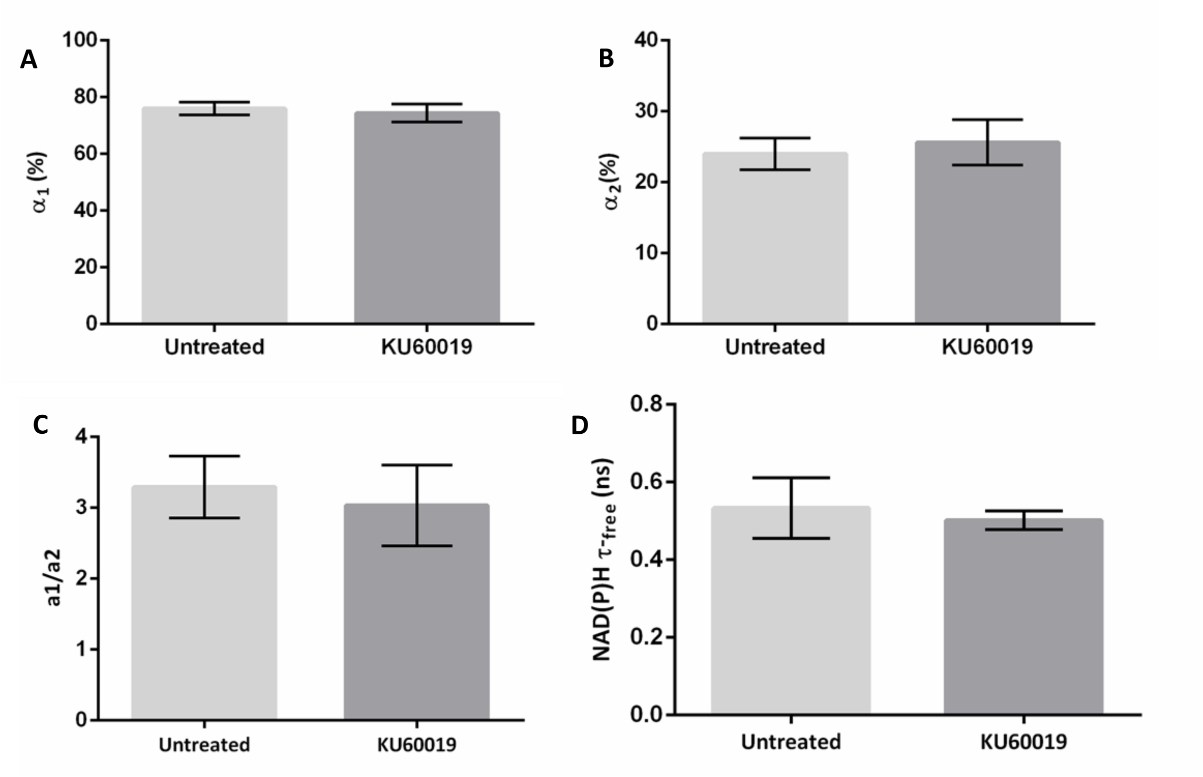

Supplementary Figure S6: **The relative fractions of free and bound NAD(P)H.** The relative fractions of free (α1, Fig S6A) and bound (α2, Fig S6B) NAD(P)H did not change significantly, nor did the ratio of free to bound NADPH (Fig S6C) between control (0.05% DMSO, n=3) and KU-treated cell cultures (n=3). The autofluorescence lifetime of τ_{free} decreased, albeit not significantly ($p=0.1634$).

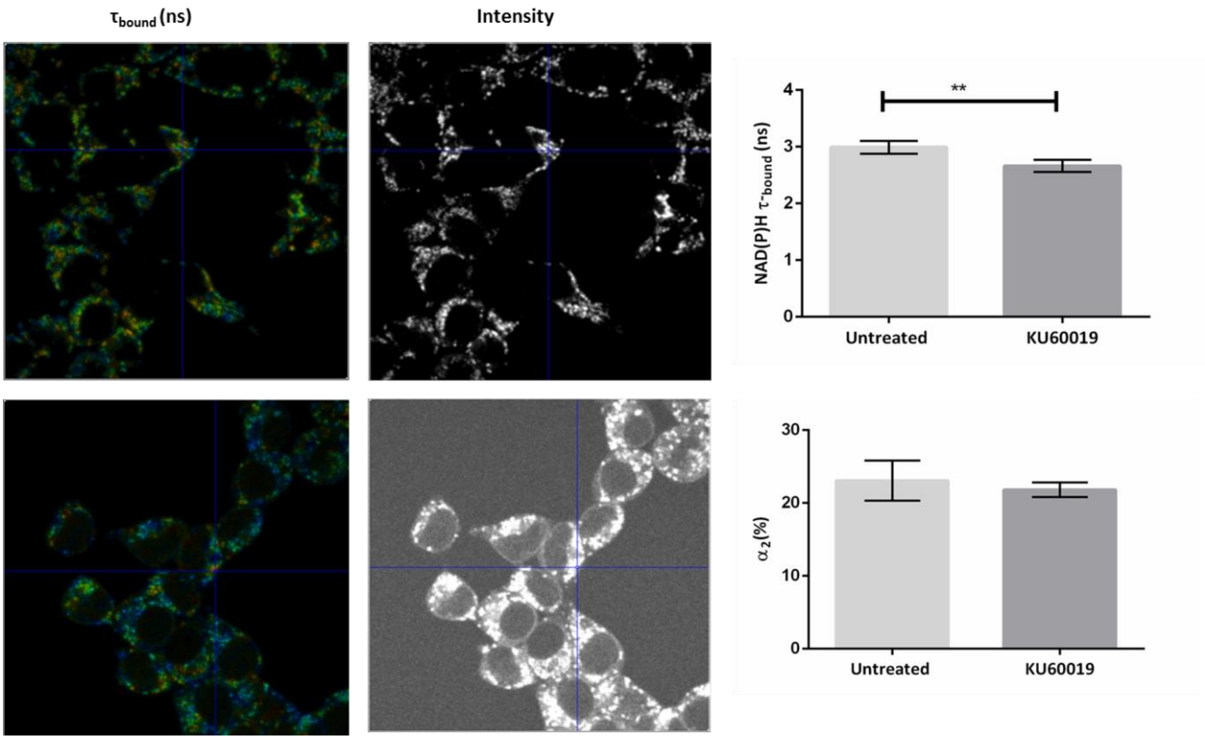

Supplementary Figure S7: Inhibition of ATM decrease NAD(P)H fluorescence decay.

Representative fluorescence decay images of HEK293 cells treated with 0.05% DMSO (vehicle control; top; n=3) or KU60019 (3 µM; n=3), which decreased NAD(P)H τ-bound lifetime (ns) significantly ($p=0.0031$, Student's t-test, mean ± s.d.), but did not influence α-bound. ** $p<0.005$

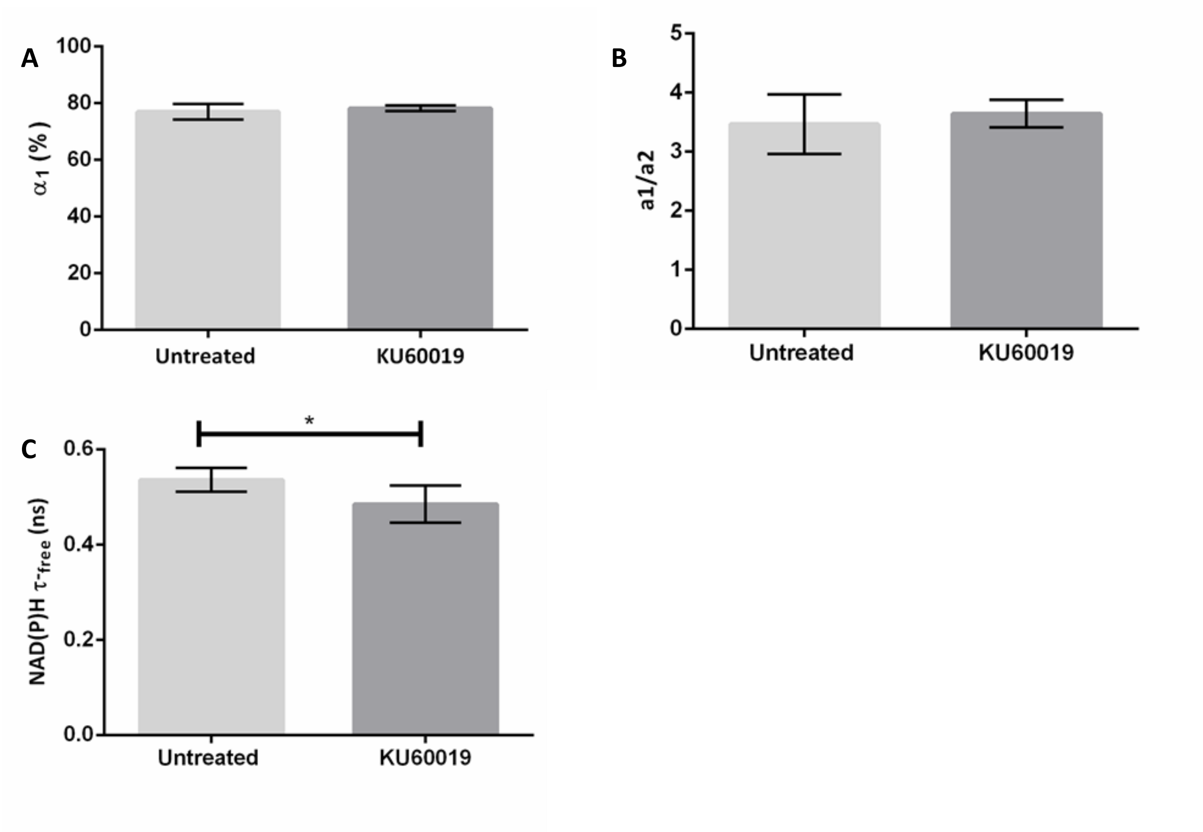

Supplementary Figure S8: The relative fractions of free and bound NAD(P)H in HEK293 cell treated with the ATM-spcific inhibitor, KU60019. The relative fractions of free (α1, Fig S8A) NAD(P)H did not change significantly, nor did the ratio of free to bound NADPH (Fig S8B) between control (n=3) and KU-treated cell cultures (n=3). The autofluorescence lifetime of τ_{free} decreased significantly when cells were treated with KU60019 compared to untreated cells (p=0.0474).

References:

1. Greenawalt, J. W. The isolation of outer and inner mitochondrial membranes. *Methods Enzymol.* **31,** 310–323 (1974).

2. Zhang, J. *et al.* Supp info: ATM functions at the peroxisome to induce pexophagy in response to ROS. *Nat. Cell Biol.* **13267,** 1–18 (2015).

3. Diokmetzidou, A. *et al.* Desmin and αB-crystallin interplay in the maintenance of mitochondrial homeostasis and cardiomyocyte survival. *J. Cell Sci.* **129,** 3705–3720 (2016).

4. Halaby, M. J., Hibma, J. C., He, J. & Yang, D. Q. ATM protein kinase mediates full activation of Akt and regulates glucose transporter 4 translocation by insulin in muscle cells. *Cell. Signal.* **20,** 1555–1563 (2008).

5. Sarkaria, J. N. *et al.* Inhibition of ATM and ATR kinase activities by the radiosensitizing agent, caffeine. *Cancer Res.* **59,** 4375–4382 (1999).

6. Blacker, T. S. *et al.* Separating NADH and NADPH fluorescence in live cells and tissues using FLIM. *Nat. Commun.* **5,** 1–9 (2014).

7. Murphy, M. P. How mitochondria produce reactive oxygen species. *Biochem. J.* **417,** 1–13 (2009).

8. Lakowicz, J. R., Szmacinski, H., Nowaczyk, K. & Johnson, M. L. Fluorescence lifetime imaging of free and protein-bound NADH. *Proc. Natl. Acad. Sci. U. S. A.* **89,** 1271–1275 (1992).

9. Blacker, T. S. & Duchen, M. R. Investigating mitochondrial redox state using NADH and NADPH autofluorescence. *Free Radic. Biol. Med.* **100,** 53–65 (2016).

10. Sharick, J. T. *et al.* Protein-bound NAD(P)H Lifetime is Sensitive to Multiple Fates of Glucose Carbon. *Sci. Rep.* **8,** 1–13 (2018).
